# Synthesis and Evaluation of the AhR Activity of Indolo[3,2*-b*]carbazole Derivatives

**DOI:** 10.3390/molecules30030690

**Published:** 2025-02-04

**Authors:** Nikitia Mexia, Stamatia Tsakou, Prokopios Magiatis

**Affiliations:** 1 Laboratory of Pharmacognosy and Natural Products Chemistry, Department of Pharmacy, National and Kapodistrian University of Athens, 15771 Athens, Greece; nmexia@pharm.uoa.gr (N.M.); tiinatsak@gmail.com (S.T.); 2 Department of Environmental Toxicology, University of California, Davis, CA 95616, USA

**Keywords:** Indolo[3,2-*b*]carbazole, 6-Formylindolo[3,2-*b*]carbazole, Aryl-hydrocarbon Receptor, *Malassezia furfur*

## Abstract

The Aryl-hydrocarbon Receptor (AhR) is implicated in the regulation of several genes, including those encoding CYP1A1. Although it is an orphan receptor, the amount of data about its relationship with skin homeostasis and nosology is constantly increasing. Interestingly, 6-formylindolo[3,2*-b*]carbazole (6-FICZ), one of the most active AhR inducers and amongst the proposed receptor’s endogenous ligands, has been detected in *Malassezia furfur* isolates from lesional skin, as well as in skin scales from patients with seborrhoeic dermatitis. Aiming to study the structure–activity relationships of the indolo[3,2*-b*]carbazole (ICZ) scaffold and to clarify if the formyl group of 6-FICZ has any specific role in AhR induction, a series of analogues of ICZ (substituted at position 6 with methyl, formyl and hydroxymethyl groups) were synthesized and evaluated for their activity on AhR in cell lines of four different species. A new simple method for the synthesis of 6-FICZ was developed. 6-Methylindolo[3,2*-b*]carbazole (6-MICZ) showed higher activity than 6-FICZ in human, rat and guinea pig cell lines, and all synthesized derivatives showed comparable activity in the mouse cell line. Therefore, the formyl group does not seem to play a significantly specific role in the affinity for AhR, and 6-FICZ seems less likely to be an endogenous ligand.

## 1. Introduction

The Aryl-hydrocarbon Receptor (AhR), or dioxin receptor as it is widely known, is a protein of 100 kDa and widely distributed in many species and tissues [1,2]. It was discovered during studies on the toxicity of TCDD, and it is implicated in cellular growth and differentiation, regulation of the expression of several genes, oxidative and anti-oxidative procedures, melanogenesis and immune responses [1,3,4,5]. It is only activated through binding with the appropriate ligands, which can be either endogenous or exogenous and natural or synthetic [6,7]. According to the literature, endogenous ligands can be compounds that are synthesized from the human body, like tryptamine and lipoxin A_4_, or compounds that are produced by the microbiota that are present on human skin and intestines, like kynurenic acid and tryptophan derivatives, including 6-Formylindolo[3,2*-b*]carbazole and an array of indolic compounds that are produced by *Malassezia* yeasts [6,8,9]. The exogenous ligands include synthetic compounds, environmental contaminants like Halogenated Aromatic Hydrocarbons (e.g., dioxin (TCDD)) and Polycyclic Aromatic Hydrocarbons (PAHs), dietary compounds, flavonoids like diosmin, alkaloids and several drugs that show high affinity for the receptor [5,6,7,8]. This leads to a cascade of transcriptional events, including the activation of caspases and the induction of the proteins of CYP450, thus stimulating the expression of genes that encode enzymes that are responsible for the metabolism of drugs and xenobiotics, such as CYP1A1 [1,10,11]. The impacts that AhR activation has in organ systems are being frequently studied, since the receptor is widely distributed in the human body. A significant part of the current research on AhR focuses on its potential physiological functions, including its participation in chemical and microbial defenses and its role in homeostasis and the modulation of the immune system in barrier organs such as the skin and the gastrointestinal tract [3]. The effects of AhR activation on cancer progress [8], intestinal homeostasis [12,13] and inflammation [8,14] have been studied extensively in recent works.

The most significant research, however, has focused on the receptor’s implication in cancer, the immune system and the inflammation process, with the data concerning the latter one being contradictory and unable to clarify whether activation of the receptor enhances or suppresses the formation of inflammation [15,16,17,18,19]. The aforementioned expressions are mediated largely by the role of AhR in oxidative stress, an active participant in the redox homeostasis and a modulator of redox signals and immune responses [20,21]. For this reason, AhR is considered as the explanation for the pathophysiology of some disorders and a potential target for new therapeutic compounds in an effort to regulate the immune response in inflammatory and autoimmune diseases [20,22]. A topical medication for the treatment of plaque psoriasis and atopic dermatitis containing an AhR agonist is already on the market [23], whilst AhR is considered a promising target for chemoprevention [24].

Even more emphasis has been placed on the relationship of AhR with skin homeostasis and nosology, with the amount of data constantly increasing. The physiological and pathological functions of skin are susceptible to AhR activation, since keratinocytes and melanocytes, as well as Langerhans’ cells, can express the receptor [25]. In healthy skin, AhR activation mediates oxidative stress, keratinocyte differentiation, skin barrier function and skin pigmentation [26]. More specifically, AhR is capable of altering melanogenesis by regulating the expression of the responsible genes in melanocytes, and hyperactivation of the receptor can cause hyperpigmentation of the skin [5]. It has also been proven to have varying roles in the pathogenesis of some skin diseases, which renders it as an attractive therapeutic target when it comes to the prevention of skin cancer or treatment of inflammatory skin diseases [26,27]. 6-Formylindolo[3,2-*b*]carbazole (6-FICZ) (**1**) is one of the most active AhR agonists and proposed by researchers as one of the receptor’s potential endogenous ligands. Research has proven that UVB radiation induces the formation of 6-FICZ (**1**) [28], although there are studies suggesting that the organism also has light-independent pathways for the production of 6-FICZ (**1**) that involve the enzymatic oxidation of tryptophan in the presence of hydrogen peroxide [29]. Its synthesis in the body consequently increases the expression of the genes that encode the enzymes of CYP450, including CYP1A1, an event that, according to previous data, is the connecting factor between AhR induction and skin carcinogenesis [5,25]. 6-FICZ (**1**) has been identified as a photosensitizer, and it seems to mediate the oxidative stress in UVA-exposed skin [30] but also to achieve photooxidative elimination of malignant cells in vitro and in vivo [31]. Additional data suggest a beneficial role for 6-FICZ (**1**) in atopic dermatitis-like skin inflammation [32].

Interestingly, 6-FICZ (**1**) has been detected by our group in skin scales from patients with seborrhoeic dermatitis, as well as in *Malassezia furfur* isolates from lesional skin, from which it was also isolated, along with other compounds that induce the receptor, e.g., tryptanthrin, indirubin and indolo[3,2-*b*]carbazole (ICZ) (**2**) [9,33].

The previously reported simultaneous detection of ICZ and 6-FICZ as *Malassezia* metabolites in human skin raised the question whether 6-FICZ is, indeed, an endogenous AhR ligand with very specific structural characteristics or just a tryptophan metabolite that is produced in the body by microorganisms or other chemical and photochemical pathways.

**Figure 1:**
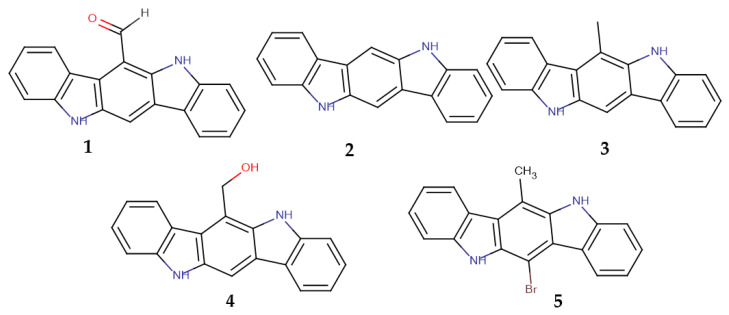
Structures of the synthesized compounds **1**–**5**.

The main aim of the current work was to clarify if the formyl group of 6-FICZ has or does not have a specific structural role in the activation of AhR, as would be expected for an endogenous ligand, or if the activity is more generally related to the indolocarbazole skeleton. For this reason, we synthesized indolocarbazoles bearing either an H (ICZ) or a formyl (6-FICZ) or a methyl (6-MICZ) or a hydroxymethyl group (6-HMICZ) at position 6 (Figure 1) and tested them under exactly the same conditions. The activity was evaluated in cell lines of four different species (human, rat, guinea pig, mouse) to investigate if there was a uniformly specific behavior of 6-FICZ in all species, as would be expected for an endogenous ligand, or if there was any variation in the activity among species.

The study of these compounds for their affinity with AhR is of high importance, since the exact physiological role of the receptor remains undetermined and ambiguous, whilst its endogenous ligand has not been positively identified to date.

## 2. Results

### 2.1. Synthesis of the Natural Products 6-FICZ (***1***) and ICZ (***2***)

**Figure 2:**
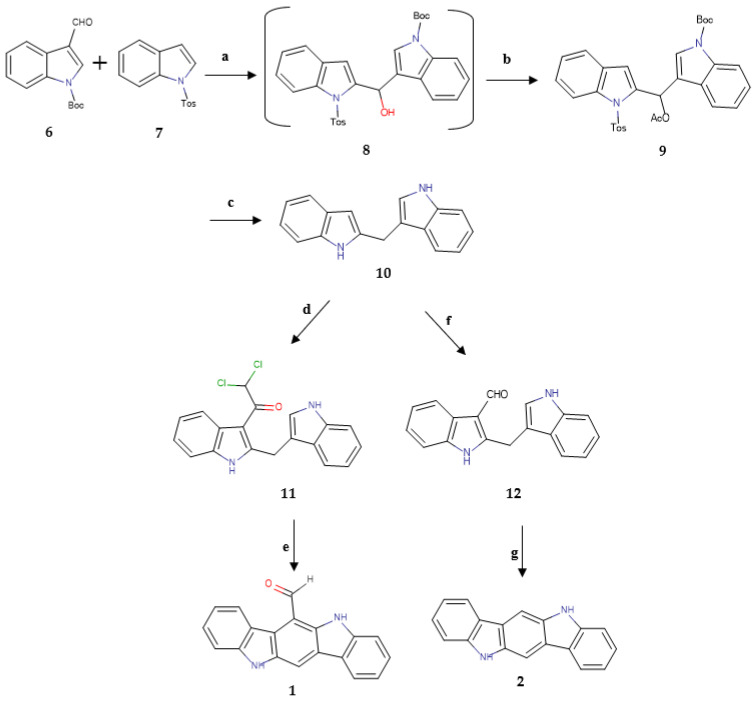
Synthesis of 6-FICZ (**1**) and ICZ (**2**). **a**: (1) t-BuLi, THF, −20 °C, (2) THF, CH_3_COCl, −20 °C to rt; **b**: Ac_2_O, pyridine, rt. 33%; **c**: Na, liquid NH_3_, THF, −78 °C. 22%; **d**: Cl_2_CHCOCl, pyridine, THF, 7 h. 39%; **e**: EtOH/HCl aq 2M (1/1), reflux, 7 h. 71%; **f**: POCl_3_, DMF, 40 °C. 30%; **g**: cat. HCl, THF, reflux. 81%.

At first, the natural compounds 6-FICZ (**1**) and ICZ (**2**) were synthesized following two different five-step syntheses with a common key intermediate, the 2,3′-methylenebisindole (**10**). The synthetic paths were based on published data [1,34], shown in Figure 2.

### 2.2. Synthesis of the Indolo[3,2-b]carbazole Derivatives ***3***–***5***

**Figure 3:**
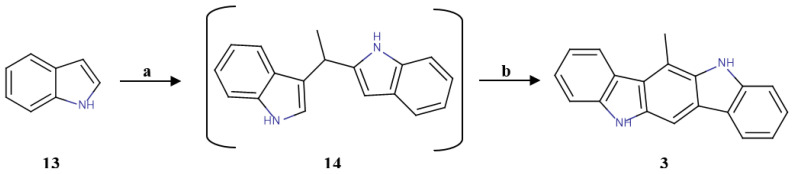
Synthesis of 6-MICZ (**3**). **a**: CH_3_CHO, I_2_, ACN, rt, 14 h; **b**: (EtO)_3_CH, CH_3_SO_3_OH, MeOH, rt, 14 h; 46% for two steps.

The synthesis of the other indolo[3,2-*b*]carbazole analogues was preceded by the one-pot formation of the compound 6-methylindolo[3,2-*b*]carbazole (6-MICZ) (**3**), according to published data [35]. This procedure consisted of an oxidative coupling between indole, acetaldehyde and triethyl orthoformate, as shown in Figure 3.

**Figure 4:**
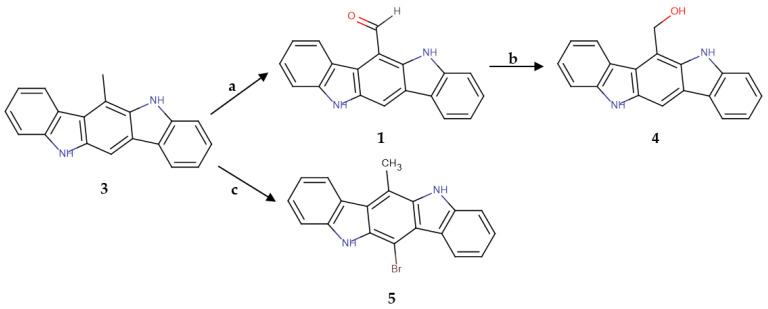
Synthesis of derivatives **4** and **5**. **a**: SeO_2_, toluene, 110–120 °C, 1 h. 10%; **b**: LiBH_4_, THF dry. 35%; **c**: NBS, pentane, CCl_4_, AIBN, 85 °C, 2 h; 25%.

The versatile methyl group at position 6 of the scaffold was a promising substrate for the application of different reactions that could lead to a variety of 6-substituted compounds. Several attempts at oxidizing the methyl of the aromatic ring in the presence of a bisubstituted amine were performed, in the most successful of which selenium dioxide was used in 3-fold excess as an oxidative means. This reaction yielded compound **1**, thus offering a new and simpler synthetic route for the formation of this important derivative. The formyl group was further reduced successfully by using borohydrides, giving the analogue **4** that bears a hydroxymethyl group at position 6 of the scaffold. Finally, the bisubstituted derivative **5** was produced via a halogenation reaction of **3** with N-bromosuccinimide. The synthetic schemes are presented in Figure 3 and Figure 4.

### 2.3. Evaluation of the Biological Activity

The activity on AhR of the synthesized compounds was evaluated in cells that were stably transfected with a luciferase reporter gene. They were tested in four different cell lines, namely in human (HG2L7.5c1), mouse (H1L7.5c3), rat (H4L7.5c2) and guinea pig (G16L7.5c1) cells. The results were calculated in EC_50_ (M) and compared to ones of dioxin (TCDD), and they are provided in Table 1.

## 3. Discussion

The main target of the current work was to investigate if the formyl group at position 6 of 6-FICZ has any structural specificity, as this would be expected if 6-FICZ was an endogenous ligand of AhR. For this purpose, we synthesized five analogues bearing the scaffold of indolo[3,2-*b*]carbazole with different substituents at position 6 of the alkaloid.

The first compounds to be synthesized were the natural products 6-FICZ (**1**) and ICZ (**2**), followed by the synthesis of the derivative that bore a methyl group at position 6 of the scaffold (6-methylindolo[3,2-*b*]carbazole (**3**)). This group was further exploited via oxidation and halogenation reactions. Several oxidation efforts led to the synthesis of the formyl derivative in only two steps and in a higher yield than the previously described method [34] that was applied at the beginning of the current work. The aldehyde of position 6 was then reduced to the corresponding hydroxymethyl group, giving product **4**. The halogenation reaction led to the formation of compound **5**, which was bisubstituted at positions 6 and 12.

The synthesized alkaloids (Figure 1) were then evaluated for their ability to induce AhR in cell lines of four different species that were stably transfected with a luciferase reporter gene. The human cell line was selected because the initial target was the investigation of the role of the substituent at position 6 of the indolocarbazole derivatives in humans. In previous studies by our group, we have shown that in many cases, the behavior of the AhR ligands is very different from species to species, as was shown to be the case for indirubins or tryptanthrins when comparing human and mouse cell lines [36]. In the current work, in addition to the commonly used mouse cell line and the human cell line, we included two more cell lines (rat and guinea pig) to investigate if there is any species specificity for the formyl group (as well as the other derivatives), or if the formyl group is the only substituent that could be well tolerated by the AhR of all species.

Although the initial synthesis of the natural products **1** and **2** was achieved through a previously described five-step synthetic pathway, as shown in Figure 1 [1,34], the overall yields for the two compounds were extremely low, reaching 2%, as some of the reactions were not standardized, with their yields varying extremely according to the conditions. Two newer syntheses of 6-FICZ (**1**) have been described in the literature [37,38], but they also involve more than two steps. On the other hand, the formation of 6-MICZ (**3**) was easy, achieved in one step and with good yield (46%) [35]. Due to these facts and because of the ability of the methyl group to be transformed into other functional groups through simple reactions, we studied analogues bearing different substituents at position 6 of the scaffold.

The initial efforts concerned the oxidation of this group, aiming to obtain a carboxylic acid group in the exact same position. The use of oxidative means like potassium permanganate and an up to 30-fold excess of selenium dioxide were not able to afford the expected analogue. However, a 3-fold excess of selenium dioxide led to the formation of 6-FICZ (**1**), providing us with a new, simpler and more efficient synthetic pathway for the formyl derivative. This path comprises only two steps, and the overall yield (5%) is significantly higher in comparison to the initial synthesis (Figure 2).

Since we managed to simplify and optimize the synthesis of 6-FICZ (**1**), we were then able to use this analogue further to acquire a compound, substituted with a hydroxymethyl group at position 6, that was very useful for the study of structure–activity relationships. Obtaining this substituent through a simple reduction of the formyl group could be easily assumed. However, this reduction was unsuccessful when performed with sodium borohydride in methanol, probably due to the participation of the aldehyde in the extended conjugation that 6-FICZ (**1**) exhibits. Thus, we figured out that a more active reagent was necessary, and the reaction was repeated with the lithium salt of borohydride. Indeed, the reduction was successful, affording the desired compound **4**.

Concerning the structure–activity relationships, it is clear that the indolo[3,2-*b*]carbazole skeleton can afford extremely active derivatives with simple modifications. Among them, 6-FICZ (**1**) and 6-MICZ (**3**) interestingly showed activity levels that were comparable to the one of TCDD in the human cell line when evaluated at 6 h with EC_50_ at 348 pM and 308 pM, respectively. However, at 24 h, the EC_50_ of TCDD remained as low as 3 × 10^−10^, but the activity of the alkaloids was much lower at the level of 10^−6^ to 10^−7^ due to differences in metabolism. Dioxin cannot be metabolized, and for this reason, the activity is retained at 24 h. On the contrary, ICZs are metabolized by the induced CYP enzymes, and for this reason, the induction of AhR is transient, with a maximum peak between 6 h and 12 h.

The activity of 6-MICZ had never been tested before, and the herein observed activity was found to be higher than that of 6-FICZ in the human, rat and guinea pig cell lines. ICZ showed lower activity than 6-FICZ in the human cell line, as previously reported [38], but higher activity in the rat and guinea pig cell lines. On the other hand, 6-HMICZ (**4**) was less active in all cases except the mouse cell line. Similarly reduced activity for 6-HMICZ has been reported by Zhang et al. [38] and Wu et al. [39], although they used different biological testing methods than the current work. The reduced activity of 6-HMICZ in comparison to 6-FICZ by more than 10 folds, as well as the similarly reduced activity of other derivatives (e.g., bearing the corresponding -COOH or -COOEt group), was used by Zhang et al. [38] as a proof for the specific role of the formyl group at position 6, stating that only the formyl group is well tolerated by the receptor. In contrast to this observation, in our hands, 6-MICZ showed higher activity than 6-FICZ in three out of four tested cell lines, and in addition, all the synthesized derivatives, independently of the substituent at position 6, showed comparable activity with 6-FICZ in the mouse receptor. These observations are in contrast with the previous structure–activity studies of Zhang et al. [38] that found a specific structural role for the formyl group and weaken the hypothesis that 6-FICZ could be an endogenous ligand of AhR.

Based on the observation of the reduced activity of 6-HMICZ, probably due to reduced lipophilicity, we proceeded in the synthesis of a less polar compound through a bromination reaction. Although the initial target was the bromination of the methyl group of 6-MICZ, the reaction of **3** with NBS led to bromination of the aromatic nucleus. The insertion of the bromine on the skeleton has a double role; it not only increases the lipophilicity of the analogue but also creates a key position that could be used in the future for the facile synthesis of other bisubstituted analogues of indolo[3,2*-b*]carbazole. As the literature has shown [35,40], it can easily react with a variety of aryl or aromatic groups. Compound **5** was included in the structure–activity study and showed an activity that was similar to that of compound **3**, confirming the general high activity of indolocarbazoles in all tested cell lines.

In conclusion, the comparable EC_50_ values of compounds **1** and **3** for the human cell line, the higher activity of **3** in other species and the lack of significant specificity among all substituents in the mouse cell line showed that the formyl group did not have any extremely specific role, as would be expected if 6-FICZ (**1**) was the receptor’s endogenous ligand (Table 1). The significant interspecies variation that was observed makes the use of the AhR of other species as models to predict activity in humans for the case of indolocarbazoles misleading.

## 4. Materials and Methods

All the solvents that were used during the experimental procedure were of analytical grade or distilled. Aluminum and glass plates, covered with silicon dioxide (Silica gel 60 F_254_), were used for TLC and preparative TLC, respectively, and the obtained chromatograms were observed in a CAMAG TLC Visualizer at 254 and 366 nm. The purification of all the synthesized compounds was performed with various chromatographic techniques such as low-pressure Column Chromatography, preparative TLC and HPLC, whilst their structure was determined with NMR 600 MHz and HRMS-LTQ Orbitrap. All the details of the known synthetic procedures and the NMR spectra of the synthesized compounds are available in the Supplementary Materials.

### 4.1. Synthesis, Purification and Structure Elucidation of Derivatives ***1***, ***4*** and ***5***

6-Formylindolo[3,2-*b*]carbazole (**1**): First, 0.063 mmol of **3** was dissolved in 800 μL toluene, and the mixture was heated at 110 °C. Then, 2.7 eq of SeO_2_ was added, and the reaction remained at the same temperature for 1h under magnetic stirring. After cooling to room temperature, the mixture was filtered under vacuum, the precipitate was washed with acetone, and the filtrate was evaporated under reduced pressure. Derivative **1** was isolated with preparative TLC (hexane:EtOAc—2:1 + 2.5% acetic acid), corresponding to a yellow zone with R_f_ = 0.67. The spectral data of the compound coincide with the bibliography [34]. The reaction yield was 10%.

6-Hydroxymethylindolo[3,2-*b*]carbazole (**4**): First, 0.033 mmol of **1** was dissolved in 3.0 mL of distilled THF, and 0.23 mmol of LiBH_4_ was added. The mixture was stirred for 1 h at 0 °C under a nitrogen atmosphere, and then, it was neutralized with the addition of acetic acid. The reaction mixture was centrifuged; the formed precipitate was collected and submitted to preparative TLC (c-hexane:EtOAc—1:1 + 1,5% acetic acid). A yellow zone with R_f_ = 0.55 was isolated, and it was identified as analogue **4**, which is also described in [39]. The reaction yield was 35%. The spectral data were comparable with the literature [39].

^1^H-NMR (MeOD, 600 MHz): δ 5.60 (s, -CH_2_-); 7.12–7.16 (2 × t overlapping, H-2/H-8, *J* = 7.3 Hz); 7.37 (t, H-3/H-9, *J* = 7.4 Hz); 7.45 (d, H-10, *J* = 7.8 Hz); 7.48 (d, H-4, *J* = 8.0 Hz); 8.03 (s, H-12); 8.10 (d, H-1, *J* = 7.7 Hz); 8.30 (d, H-7, *J* = 8.0 Hz).

6-Methyl-12-bromoindolo[3,2-*b*]carbazole (**5**): First, 0.063 mmol of **3** was dissolved in 100 μL pentane, followed by the addition of NBS (0.96 mmol), CCl_4_ (1 mL) and a catalytic amount of AIBN. The mixture was heated at 85 °C for 2 h and, after cooling at room temperature, filtered. The formed precipitate was washed with CCl_4,_ and the filtrate was evaporated under reduced pressure. A yellow zone with R_f_ = 0.75 was isolated with preparative TLC (hexane:EtOAc—2:1) and identified as the bisubstituted analogue 5 after comparison with bibliographic data [35]. The yield was 25%.

^1^H-NMR (DMSO-d_6_, 400MHz): δ 3.04 (s, -CH_3_); 7.21 (t, H-2 and H-8, *J* = 7.6 Hz); 7.41–7.50 (m, H-3 and H-9); 7.56 (d, H-10, *J* = 7.8 Hz); 7.60 (d, H-4, *J* = 7.9 Hz); 8.27 (d, H-7, *J* = 7.9 Hz); 8.69 (d, H-1, *J* = 7.5 Hz); 11.14 (s, -NH); 11.30 (s, -NH).

### 4.2. Protocol for the Evaluation of AhR Activity

The synthesized alkaloids were evaluated for their ability to induce AhR in four different cell lines, more specifically in human (HG2L7.5c1), mouse (H1L7.5c3), rat (H4L7.5c2) and guinea pig (G16L7.5c1) cells that were stably transfected with a luciferase reporter gene [41]. The results were calculated in EC_50_ (M) and compared to those of dioxin (TCDD), the principal ligand of the receptor. The protocol followed is described below.

Human, mouse, rat and guinea pig cells were grown in 100 mm cell culture plates (Corning Glass Works; Corning, NY, USA) using a sterile technique, maintained in alpha-minimum essential media (α-MEM) supplemented with 10% (*v*/*v*) fetal bovine serum (FBS) and G418 (1 mg/mL), and incubated at >80% humidity and 37◦C. For the CALUX analysis, plates of stable cell clones (approximately 80–100% confluent) were trypsinized and resuspended in 20 mL α-MEM. An aliquot (100 μL) of the indicated cell suspension was added into sterile 96-well tissue culture plates (Corning), and the plates were incubated for 24 h prior to ligand exposure, allowing the cells to reach confluence. Prior to ligand addition, the wells were washed with 1 × phosphate-buffered saline (PBS), and then, cells were incubated with the indicated concentration of DMSO (10 μL/mL) or corresponding solutions of TCDD or tested compounds in DMSO for 6 h or 24 h at 37 °C. After incubation, the cells were washed twice with PBS, 100 μL of 1 × Lysis buffer (Promega; Madison, WI, USA) was added to each well, and the plate was placed on a plate shaker at room temperature until the cells were lysed (approximately 20 min). Luciferase activity was measured using an automated microplate luminometer (Anthos Lucy2, Anthos Labtec Instruments, Wals/Salzburg, Austria) in enhanced flash mode with the automatic injection of 50 μL of Promega-stabilized luciferase reagent. Examples of the induction curves of the tested compounds are available in the Supplementary Materials.

## 5. Conclusions

The current study presents a new simple synthesis of 6-FICZ in two steps and, in addition, confirms that indolocarbazole is a scaffold of some of the most active AhR inducers that have ever been reported. The fact that 6-MICZ was more active than 6-FICZ in the human, rat and guinea pig cell lines, and the fact that all the synthesized derivatives had similar activity levels in the mouse cell line, implies that the formyl group of 6-FICZ does not have any specific structural role, as would be expected for an endogenous ligand; rather, AhR induction seems to be a more general characteristic of the indolocarbazole scaffold. Based on this observation, it seems less likely that 6-FICZ is an endogenous ligand; however, this needs further studies. On the other hand, the high activity levels of all the studied compounds suggest that compounds bearing the indolocarbazole scaffold can be potential drug candidates for the treatment of inflammatory conditions, cancer or neurological disorders [42,43]. Finally, the observed significant interspecies variation in the AhR activation for the same compound confirms the previous observation that activity measured in other species cannot be safely used to predict the activity in the human AhR, also for the case of indolocarbazoles.

## Figures and Tables

**Table 1:** EC_50_s (in M) of derivatives **1**–**5** in four different cell lines.

Compound	EC_50_, 6 h Human	EC_50_, 6 h Rat	EC_50_, 6 h Guinea Pig	EC_50_, 6 h Mouse
**1**	3.48 × 10^−10^	7.99 × 10^−11^	5.77 × 10^−10^	4.82 × 10^−10^
**2**	6.40 × 10^−10^	9.50 × 10^−12^	1.74 × 10^−10^	9.80 × 10^−10^
**3**	3.08 × 10^−10^	5.88 × 10^−11^	9.13 × 10^−11^	9.10 × 10^−10^
**4**	4.52 × 10^−9^	4.73 × 10^−10^	5.77 × 10^−9^	9.22 × 10^−10^
**5**	3.28 × 10^−10^	5.98 × 10^−11^	9.93 × 10^−11^	9.20 × 10^−10^
TCDD	5.7 × 10^−10^	5.9 × 10^−11^	1.0 × 10^−10^	1.0 × 10^−10^

## Data Availability

The original contributions presented in this study are included in the article/Supplementary Materials. Further inquiries can be directed to the corresponding author.

## Supplementary Materials

The following supporting information can be downloaded at https://www.mdpi.com/article/10.3390/molecules30030690/s1: Synthetic procedures, NMR spectra of compounds **1–12** and AhR induction curves of the tested compounds.

## Abbreviations

The following abbreviations are used in this manuscript:

AhR	Aryl-hydrocarbon Receptor
CYP1A1	Cytochrome Ρ450, 1A proteins
6-FICZ	6-Formylindolo[3,2-*b*]carbazole
ICZ	Indolo[3,2-*b*]carbazole
6-MICZ	6-Methylindolo[3,2-*b*]carbazole
EC_50_	Half maximal Effective Concentration
TCDD	Dioxin
PAHs	Polycyclic Aromatic Hydrocarbons
CYP450	Cytochrome Ρ450
6-HMICZ	6-Hydroxymethylindolo[3,2*-b*]carbazole
