# Supplementary material for: Synthesis and Evaluation of the AhR Activity of Indolo[3,2-b]carbazole Derivatives
Source: Molecules. 2025 Feb 4;30(3):690. doi: 10.3390/molecules30030690 (PMC11820409; doi:10.3390/molecules30030690)
Supplement: Supplementary file 1 [file molecules-30-00690-s001.zip › molecules-3426405-supplementary.pdf]

Supplementary material
Synthesis and Evaluation of the AhR activity of Indolo[3,2-*b*]carbazole Derivatives

Nikitia Mexia, Stamatia Tsakou, and Prokopios Magiatis

Synthesis of 6-FICZ (1) and ICZ (2)

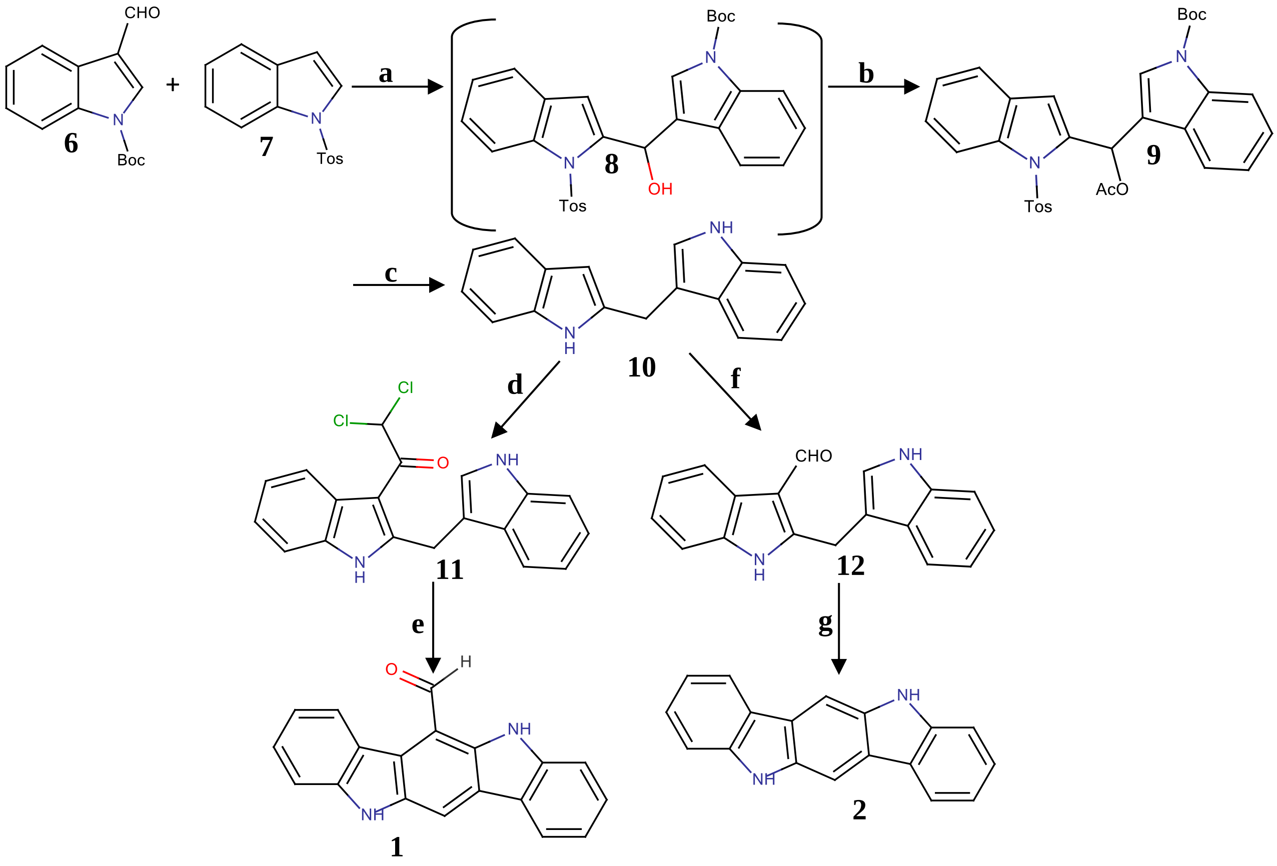

a: 1) t-BuLi, THF, -20°C, 2) THF, CH_3COCl, -20°C to rt; **b**: Ac_2O, pyridine, rt; **c**: Na, liquid NH_3, THF, -78°C; **d**: $Cl_2CHCOCl$, pyridine, THF, 7h; **e**: EtOH/HCl aq 2M (1/1), reflux, 7h; **f**: $POCl_3$, DMF, 40°C, **g**: cat. HCl, THF, reflux

Synthesis of 3-[Hydroxy-(1-phenylsulfonyl-1H-indol-2-yl)-methyl}-indole-1-carboxylic acid tert-butyl ester (8) [2]: 4.98mmol of 1-phenylsulfonyl-1H-indole **(7)** were dissolved in 42 mL dry THF and the mixture was cooled at -20°C under argon. Then 3.44 mL n-BuLi (1.6M in hexane) were added and the mixture was stirred at -5°C for 40 min and at room temperature for 5 more minutes. The mixture was again cooled at -20°C and a solution of 5.01 mmol of 3-Formylindole-1-carboxylic acid tert-butyl ester **(6)** in 18mL dry THF was added. The mixture was stirred at -20°C for 2h, followed by the addition of 9.5 mmol of acetyl chloride and it was allowed to stir overnight at room temperature. Afterwards, 25mL of distilled water were added and the mixture was extracted with diethyl ether, then washed with a 5% aqueous solution of sodium bicarbonate and water and dried over sodium sulfate. Both the non-acylated **8** and the acylated **9** compounds were isolated by column chromatography with a mixture of cyclohexane and ethyl acetate 95:5. The yields for this reaction were 17% for **8** and 16% for **9**.
Yellow solid, MW=502.63g/mol, R_f=0.27 (hexane:EtOAc – 5:1)

<u>^{1}H-NMR (Acetone-d_6, 600MHz)</u>: δ 1.66 (s, 9H); 5.16 (d, 1H, *J*=5.8Hz); 6.84 (d, 1H, *J*=5.8Hz); 7.16 (t, 1H, *J*=7.9Hz); 7.24 (t, 1H, *J*=7.9Hz); 7.31 (d, 1H, *J*=7.9Hz); 7.35 (m,2H); 7.44 (d, 1H, *J*=7.9Hz); 7.47 (m, 3H); 7.53 (d, 1H, *J*=7.9Hz); 7.64 (t, 1H, *J*=7.9Hz); 7.87 (d, 2H, *J*=7.9Hz); 8.13 (d, 1H, *J*=7.9Hz); 8.17 (d, 1H, *J*=7.9Hz)

Synthesis of 3-[Acetoxy-(1-phenylsulfonyl-1H-indol-2-yl)-methyl}-indole-1-carboxylic acid tert-butyl ester (9) [2]: 0.84mmol of **8** were dissolved in 1 mL of pyridine and 1 mL of acetic anhydride and the mixture was stirred under argon for 24 h. The reaction mixture was concentrated under vacuum and the total yield was 33%.
Yellow solid, MW=544.67g/mol, R_f=0.33 (hexane:EtOAc – 5:1)
^{1}H-NMR (Acetone-d_6, 600MHz): δ 1.65 (s, 9H); 2.14 (s, 3H); 6.94 (brs, 1H); 7.23 (td, 1H, *J*=8.0Hz, 1Hz); 7.27 (td, 1H, *J*=8.0Hz, 1.0Hz); 7.36 (tt, 2H, *J*=7.9Hz, 1.3Hz); 7.51 (m, 3H); 7.56 (d, 2H, *J*=8.0Hz); 7.66 (tt, 1H, *J*=7.9Hz, 1.3Hz); 7.84 (dd, 2H, *J*=8.0Hz, 1.0Hz); 7.87 (s, 1H); 8.12 (dd, 1H, *J*=8.0Hz, 1.0Hz); 8.14 (d, 1H, *J*=8.0Hz)

Synthesis of 2,3′-Methylenebisindole (10) [2]: Gas ammonia was liquified at -78°C until the collection of approx. 25 mL. A solution of 1.63 mmol of **9** in 3 mL dry THF was added along with small pieces of sodium until the mixture remained blue for 30min. 2g of ammonium formate were then added and the mixture was allowed to warm up at room temperature. Afterwards, 30 mL of a 10% aqueous solution of ammonium formate were added and the reaction was extracted with diethyl ether, then washed water and dried over sodium sulfate. The product was isolated by column chromatography with a mixture of cyclohexane and ethyl acetate ranging from 95:5 to 92.5:7.5. The yield was 22%.
White solid, MW=246.33g/mol, R_f=0.52 (hexane:EtOAc – 3:1)
^{1}H-NMR (Acetone-d_6, 600MHz): δ 4.27 (s, 2H); 6.26 (q, 1H, *J*=1.0Hz); 6.92 (td, 1H, *J*=8.0Hz, 1.0Hz); 6.95 (td, 1H, *J*=8.0Hz, 1.0Hz); 6.97 (td, 1H, *J*=8.1Hz, 1.0Hz); 7.07 (td, 1H, *J*=8.1Hz, 1.0 Hz); 7.22 (t, 1H, *J*=1.0Hz); 7.25 (dd, 1H, *J*= 8.0Hz, 1.0Hz); 7.38 (dt, 1H, *J*= 8.1Hz, 1.0Hz); 7.42 (dd, 1H, *J*=8.1Hz, 1.0Hz); 7.50 (dd, 1H, *J*=8.0Hz, 1.0Hz); 9.95 (brs, 1H); 10.09 (brs, 1H)

Synthesis of 2,2-dichloro-1-[2-(1H-indol-3-ylmethyl)-1H-indol-3-yl)-ethanone (11) [1]: 0.32 mmol of **10** was dissolved in 3.6 mL of dry THF under argon in a round bottom flask covered with aluminum foil. 31 μL of dry pyridine were added and the solution was cooled at 0°C, followed by the dropwise addition of 0.4 mmol of 2,2-dichloroacetyl chloride. The mixture was stirred at room temperature for 7 h and 20 mL of dichloromethane were added subsequently. The mixture was extracted first with 10 mL of HCl 2M and then with 10 mL of a 10% aqueous solution of sodium bicarbonate. The organic phase was washed with water and dried over sodium sulfate. The product was isolated by column chromatography with a mixture of cyclohexane and ethyl acetate ranging from 95:5 to 80:20. The resulting product is air- and light-sensitive [1] and the yield was 39%.
Brown solid, MW=357.23g/mol, R_f=0.63 (hexanes:EtOAc – 1:1)
^{1}H-NMR ($CDCl_3$, 600MHz): δ 4.77 (s, 2H); 6.90 (s, 1H); 7.14 (t, 1H, *J*=7.6Hz); 7.20 (m, 2H); 7.30 (m, 3H); 7.44 (d, 1H, *J*=7.9Hz); 7.48 (d, 1H, *J*=8.2Hz); 7.89 (d, 1H, *J*=8.1Hz); 8.33 (s, -NH); 8.57 (s, -NH)

<u>**Synthesis of 6-Formylindolo[3,2-*b*]carbazole (1) [1]**</u>:

45.0 mg of **11** were dissolved in 4 mL of solvent mixture containing ethanol and aqueous solution HCl 2M in a 1:1 ratio. The mixture was brought to boiling point (103°C) and was stirred for 7h. After it was cooled down to room temperature, the remaining solvent was evaporated until the liquid was colorless and the brown precipitate was filtered and dried. The yield of the reaction was 71%.

Yellow solid, MW=284.33g/mol, R_f=0.66 (c-hexane:EtOAc – 1:1), UV: λ_{max} (nm) 248, 332, 390
^{1}H-NMR (DMSO-d_6, 600MHz): δ 7.19 (t, H-2, *J*=7.6Hz); 7.22(t, H-8, *J*=7.4Hz); 7.43 (t, H-9, *J*=7.4Hz); 7.47 (t, H-3, *J*=7.6Hz); 7.58 (d, H-10, *J*=8.1Hz); 7.74 (d, H-4, *J*=8.1Hz); 8.29 (d, H-1, *J*=7.7Hz); 8.56 (d, H-7, *J*=8,1Hz); 8.60 (s, H-12); 11.36 (s, -CHO); 11.64 (s, -NH); 11.75 (s, -NH)
^{13}C NMR (150 MHz, DMSO- d_6): δ 190.5, 142.1, 142.1, 135.8, 135.2, 126.9, 126.7, 125.1, 123.8, 122.1, 121.8, 121.5, 120.9, 119.7, 119.3, 112.8, 112.5, 111.9, 110.4

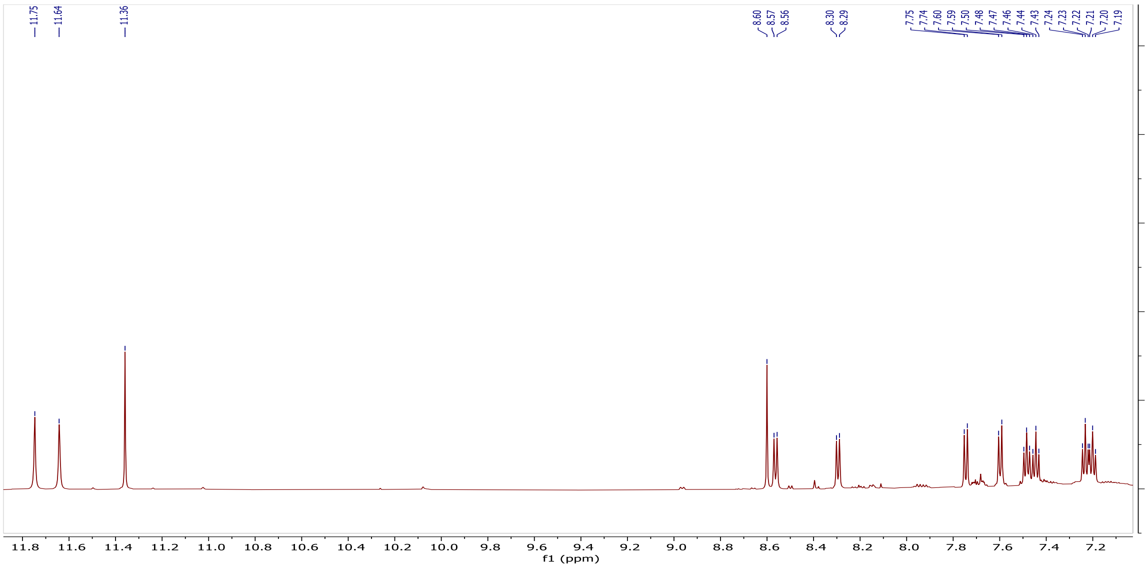

^{1}H-NMR of **1**, DMSO-d_6, 600MHz

Synthesis of Malassezin (12) [2]: A round bottom flask with 1mL of dry DMF was stirred at 0°C under argon for 5 minutes, then 36 μL of phosphorus oxychloride were added and the solution was stirred at 0°C for 20 more min. Afterwards, the mixture was stirred at room temperature for 15 minutes further, then it was cooled again at 0°C followed by the addition of a solution of 0.35mmol of **10** in 1.5mL dry DMF and it was further stirred for 20 minutes. The mixture was then carefully warmed at 40°C and the progress of the reaction was followed by TLC. Upon completion, 12 mL of a 10% aqueous solution of sodium bicarbonate were added and the mixture was extracted with diethyl ether. The organic phase was washed with an aqueous solution of NaOH 1M, then with water and dried over sodium sulfate. The product was isolated by column chromatography with a mixture of cyclohexane and ethyl acetate ranging from 75:25 to 70:30. The resulting product is air- and light- sensitive [1] and the yield was 30%.
Pink solid, MW=274.34g/mol, R_f=0.49 (hexane:EtOAc – 1:1)
^{1}H-NMR (Acetone-d_6, 600MHz): δ 4.69 (s, -CH_2); 6.98 (td, 1H, *J*=7.9Hz, 1.0Hz); 7.10 (t, 1H, *J*=7.1Hz); 7.16 (m, 2H); 7.31 (d, 1H, *J*=2.2Hz); 7.34 (dd, 1H, *J*=7.6Hz, 1.0Hz); 7.41 (d, 1H, *J*=8.2Hz); 7.51 (d, 1H, *J*=7.9Hz); 8.20 (d, 1H, *J*=7.1Hz); 10.23 (s, -NH); 10.40 (s, -CHO); 10.83 (s, -NH)
Synthesis of Indolo[3,2-*b*]carbazole (2) [2]: One drop of fuming HCl (37%) was added in a solution of 0.031mmol of **12** in 10mL THF. The mixture was brought to boiling point (90°C) and it was stirred under argon overnight. Afterwards, the mixture was cooled to room temperature and the precipitating yellowish solid was filtered. The precipitate was further purified with centrifugation after addition of acetone. The reaction yield was 81%.
Yellow solid, MW=256.32g/mol, R_f=0.49 (MeOH 100%, RP-18), UV: λ_{max} (nm) 271, 333, 398
^{1}H-NMR (DMSO-d_6, 600MHz): δ 7.12 (td, H-2/8, *J*=7.8Hz, 1Hz); 7.37 (td, H-3/9, *J*=8.0Hz, 1Hz); 7.45 (d, H-4/10, *J*=8.0Hz); 8.11 (s, H-6/12); 8.19 (d, H-1/7, *J*=7.8Hz); 11.00 (brs, 2x-NH)

^{13}C NMR (150 MHz, DMSO- d_6): δ 142.0 (2C), 135.9 (2C), 126.3 (2C), 123.5 (2C), 123.4 (2C), 121.1 (2C), 118.5 (2C), 111.3 (2C), 101.3 (2C)

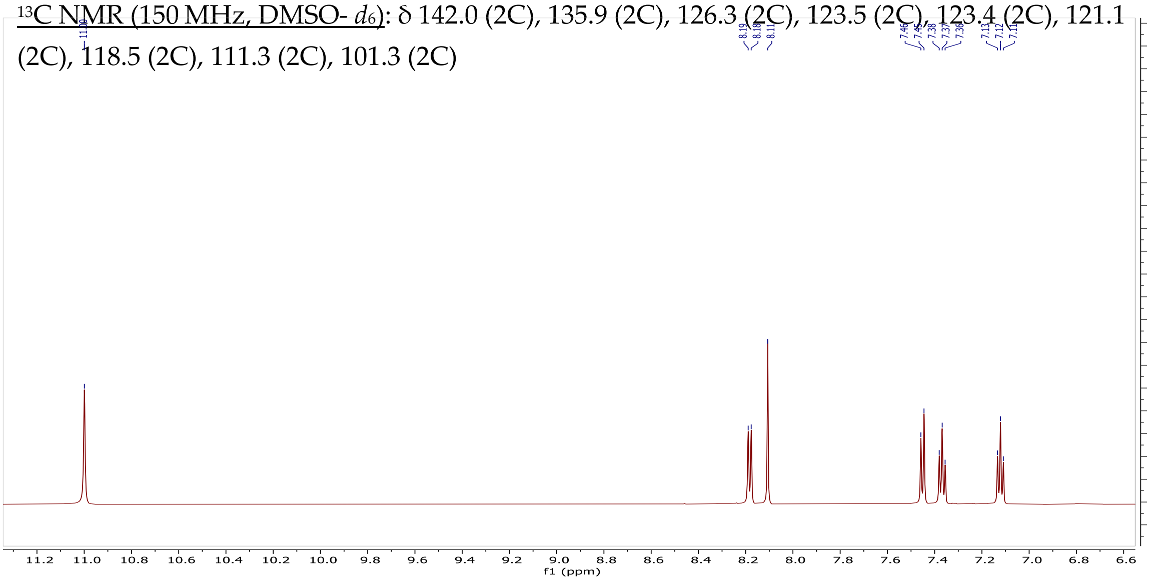

^{1}H-NMR of **2**, DMSO-d_6, 600MHz

Synthesis of 6-MICZ (3)

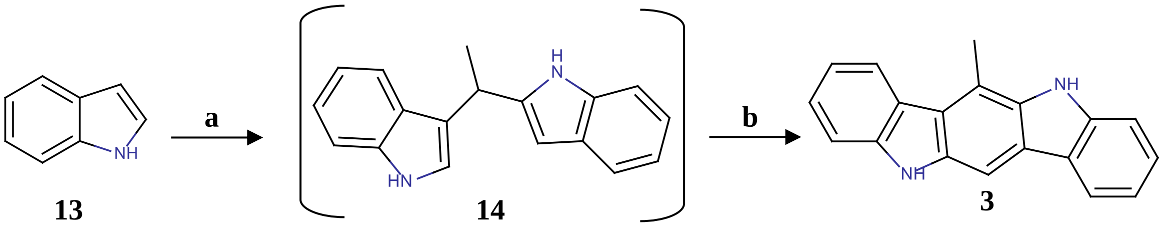

a: CH_3CHO, I_2, ACN, rt, 14h; **b:** $(EtO)_3CH$, CH_3SO_3OH, MeOH, rt, 14h

6-Methylindolo[3,2-*b*]carbazole (3) [3]: 3.67 mmol of indole **(13)** were dissolved in 5 mL of acetonitrile followed by the addition of 100 μL of an aqueous solution of formaldehyde 37% in H_2O (1.8mmol) και 3.67 mmol iodine. The mixture was stirred at room temperature for 14 h and then 10 mL of a saturated aqueous solution of sodium sulfite were added. The mixture was extracted with ethyl acetate and dried over sodium sulfate. The product **14** was a brown precipitate that was used to the next step without any further treatment as it is highly unstable. 3.6 mmol of **14** were dissolved in 2 mL of MeOH followed by the addition of 1.8 mmol of triethyl orthoformate and 0.37 mmol of methanesulfonic acid as catalyst. A red precipitate was formed immediately after the addition of the catalyst and the mixture was stirred for 14h. Afterwards, the precipitate was filtered and dissolved in methanol. The product was isolated by column chromatography with a mixture of cyclohexane and ethyl acetate, 98:2. The yield was 46%.
White precipitate, MW=270.33g/mol, R_f=0.78 (c-hexane:EtOAc – 1:1)
^{1}H-NMR (DMSO-d_6, 600MHz): δ 3.04 (s, -CH_3); 7.12 (t, H-2, *J*=7.6Hz); 7.14(t, H-8, *J*=7.7Hz); 7.37 (t, H-3 & H-9, *J*=7.6Hz); 7.47 (d, H-10, *J*=7.6Hz); 7.48 (d, H-4, *J*=7.6Hz); 7.96 (s, H-12); 8.17 (d, H-1, *J*=7.6Hz); 8.24 (d, H-7, *J*=7.7Hz); 10.95 (s, -NH); 11.06 (s, -NH)

<u>^{13}C NMR (150 MHz, DMSO- d_6)</u>: δ 142.0, 142.0, 135.9, 134.9, 126.0, 125.4, 123.4, 123.3, 122.5, 121.4, 120.9, 118.3, 118.2, 113.6, 111.2, 110.8, 98.5, 15.2

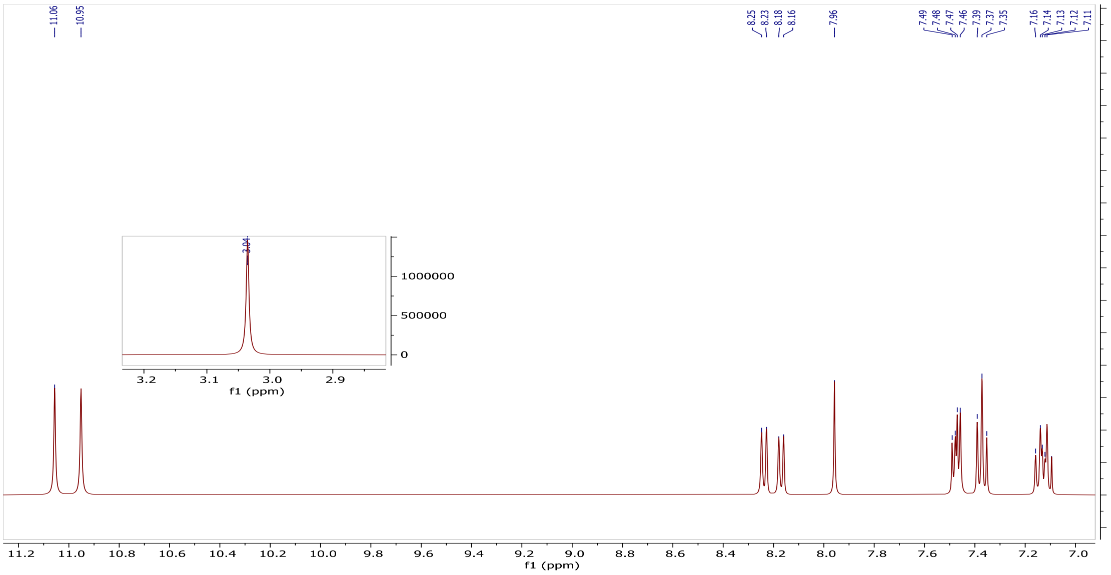

^{1}H-NMR of **3**, DMSO-d$_6$, 600MHz

Synthesis of 4 and 5

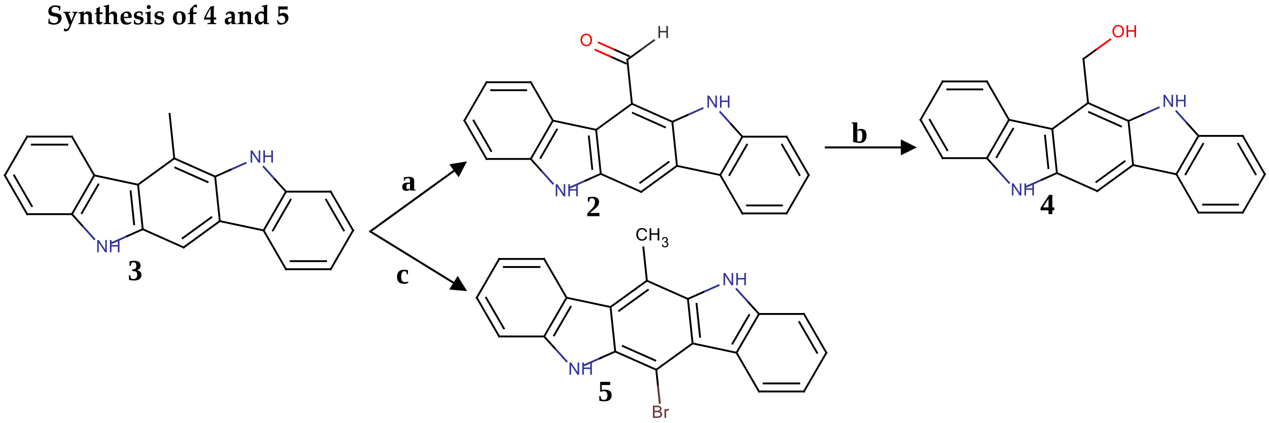

a: SeO_2, toluene, 110-120°C, 1h; **b:** $LiBH_4$, THF dry; **c**: NBS, pentane, CCl_4, AIBN, 85°C, 2h

<u>**6-Hydroxymethylindolo[3,2-*b*]carbazole (4)**</u>:

The reaction procedure is described in the main article.

Yellow solid, MW=286.33g/mol, R_f=0.55 (c-hexane:EtOAc – 1:1 + 1.5% Acetic Acid).

<u>^{1}H-NMR (MeOD, 600MHz)</u>: δ 5.60 (s, -CH_2-); 7.12-7.16 (2×t overlapping, H-2/H-8, *J*=7.3Hz); 7.37 (t, H-3/H-9, *J*=7,4Hz); 7.45 (d, H-10, *J*=7.8Hz); 7.48 (d, H-4, *J*=8.0Hz); 8.03 (s, H-12); 8.10 (d, H-1, *J*=7.7Hz); 8.30 (d, H-7, *J*=8.0Hz)

^{13}C-NMR (MeOD, 150MHz): δ 142.2, 142.1, 136.4, 134.8, 126.2, 126.0, 124.3, 123.8, 123.4, 123.4, 121.2, 120.7, 118.9, 118.8, 117.5, 111.0, 111.0, 100.6, 59.5 (-CH_2-)

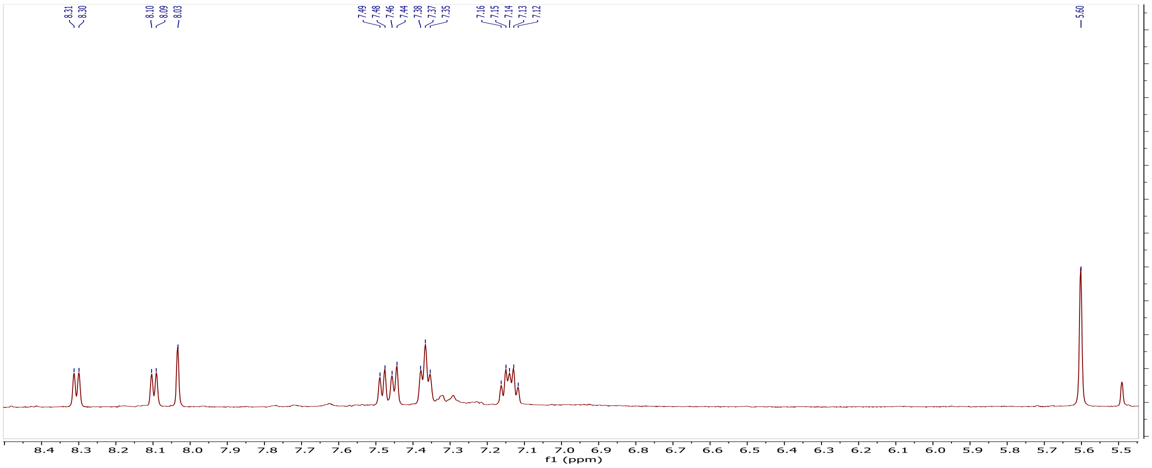

^{1}H-NMR of **4**, MeOD, 600MHz

6-Methyl-12-bromoindolo[3,2-*b*]carbazole (5):

The reaction procedure is described in the main article.

Yellow solid, MW=349.32g/mol, R_f=0.75 (c-hexane:EtOAc – 2:1)

^{1}H-NMR (DMSO-d_6, 400MHz): δ 3.04 (s, -CH_3); 7.21 (t, H-2 & H-8, *J*=7.6Hz); 7.41-7.50 (m, H-3 & H-9); 7.56 (d, H-10, *J*=7.8Hz); 7.60 (d, H-4, *J*=7.9Hz); 8.27 (d, H-7, *J*=7.9Hz); 8.69 (d, H-1, *J*=7.5Hz); 11.14 (s, -NH); 11.30 (s, -NH)

^{13}C NMR (100 MHz, DMSO- *d_6*): δ 141.6, 141.6, 134.5, 134.0, 126.1, 125.6, 122.9, 122.1, 121.7, 120.9, 119.9, 118.3, 118.0, 113.8, 111.4, 110.8, 93.0, 15.0

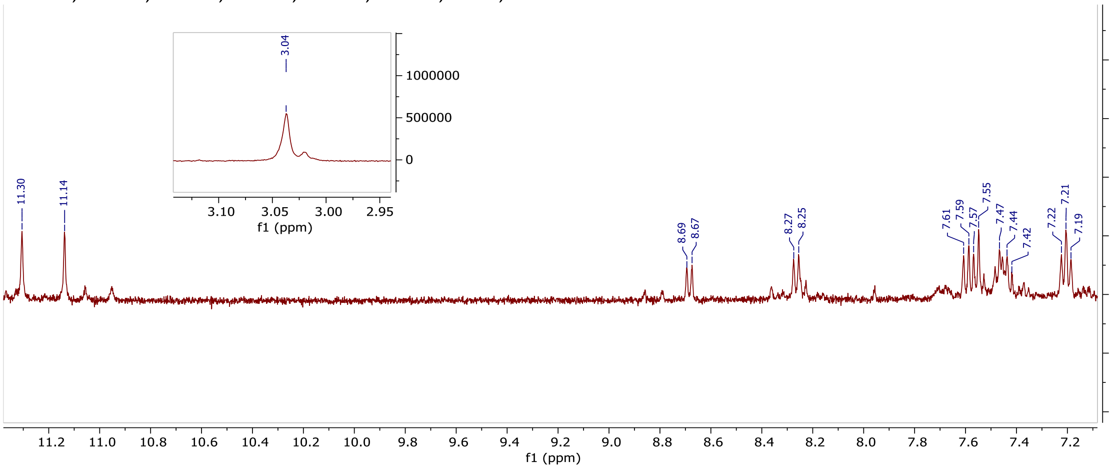

^{1}H-NMR of (**5**), DMSO-d_6, 600MHz

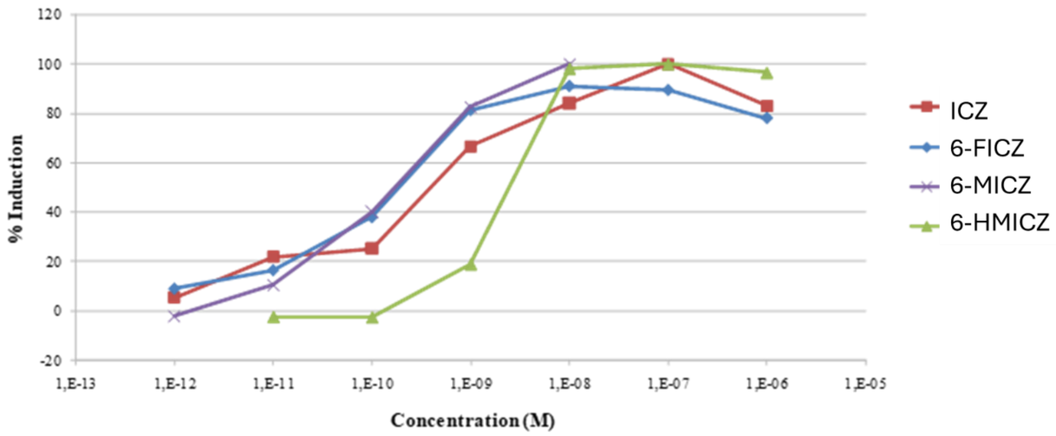

Examples of the % induction of AhR by ICZs in the human cell line. 100% induction was achieved with TCDD

References

1. Tholander J., Bergman J. **(1999)** Syntheses of 6-substituted Indolo[3,2-b]carbazoles, including 6-formylindolo[3,2-b]carbazole, an extremely efficient ligand for the TCDD (Ah) Receptor, *Tetrahedron*, 55, 6243-6260

2. Wille G., Mayser P., Thoma W. *et al.* Malassezin-a novel agonist of the Arylhydrocarbon Receptor from the yeast *Malassezia furfur*, *Bjorg. Med. Chem.*, **2001**, *9*, 955-960

3. Gu R., Hameurlaine A., Dehaen W. **(2007)** Facile One-Pot Synthesis of 6-Monosubstituted and 6,12-Disubstituted 5,11-Dihydroindolo[3,2-*b*]carbazoles and Preparation of Various Functionalized Derivatives. *J. Org. Chem.*, 72, 7207-7213
